# Macroalgae in biomonitoring of metal pollution in the Bay of Bengal coastal waters of Cox’s Bazar and surrounding areas

**DOI:** 10.1038/s41598-021-99750-7

**Published:** 2021-10-25

**Authors:** Md.Refat Jahan Rakib, Y. N. Jolly, Diana Carolina Dioses-Salinas, Carlos Ivan Pizarro-Ortega, Gabriel Enrique De-la-Torre, Mayeen Uddin Khandaker, Abdullah Alsubaie, Abdulraheem S. A. Almalki, D. A. Bradley

**Affiliations:** 1 Department of Fisheries and Marine Science, Faculty of Science, Noakhali Science and Technology University, Noakhali, Bangladesh; 2 Atmospheric and Environmental Chemistry Laboratory, Atomic Energy Centre, Dhaka, 1000 Bangladesh; 3 Universidad San Ignacio de Loyola, Av. La Fontana 501, Lima 12, Lima, Peru; 4 Centre for Applied Physics and Radiation Technologies, School of Engineering and Technology, Sunway University, 47500 Bandar Sunway, Selangor Malaysia; 5 Department of Physics, College of Khurma, Taif University, P.O. Box 11099, Taif, 21944 Saudi Arabia; 6 Department of Chemistry, Faculty of Science, Taif University, Taif, 21974 Saudi Arabia; 7 Department of Physics, University of Surrey, Guildford, GU2 7XH UK

**Keywords:** Biogeochemistry, Environmental sciences, Ocean sciences

## Abstract

Although coastal water marine algae have been popularly used by others as indicators of heavy metal pollution, data within the Bay of Bengal for the estuarine Cox’s Bazar region and Saint Martin’s Island has remained scarce. Using marine algae, the study herein forms an effort in biomonitoring of metal contamination in the aforementioned Bangladesh areas. A total of 10 seaweed species were collected, including edible varieties, analyzed for metal levels through the use of the technique of EDXRF. From greatest to least, measured mean metal concentrations in descending order have been found to be K > Fe > Zr > Br > Sr > Zn > Mn > Rb > Cu > As > Pb > Cr > Co. Potential toxic heavy metals such as Pb, As, and Cr appear at lower concentration values compared to that found for essential mineral elements. However, the presence of Pb in *Sargassum oligocystum* species has been observed to exceed the maximum international guidance level. Given that some of the algae species are cultivated for human consumption, the non-carcinogenic and carcinogenic indices were calculated, shown to be slightly lower than the maxima recommended by the international organizations. Overall, the present results are consistent with literature data suggesting that heavy metal macroalgae biomonitoring may be species-specific. To the best of our knowledge, this study represents the first comprehensive macroalgae biomonitoring study of metal contamination from the coastal waters of Cox’s Bazar and beyond.

## Introduction

Marine pollution began to be recognized and taken on importance since 1950 due to its consequences on human health through the intake of contaminated seafood/foodstuffs^1^. In fact, oceanic habitats are subject to a wide variety of pollutants, among them, heavy metals and trace elements took a significant position^2^. In recent decades, the marine environment shows increasing concentrations of these pollutants following large-scale urbanization, industrialization, and greater agricultural activities^3,4^. Heavy metals and metalloids from anthropogenic activities, including mining, milling, petrochemicals processing, electronics industry, and municipal waste, directly discharged into the marine environment or transported into the greater aquatic system via estuaries are of particular concern^5^. Particularly, heavy metals are transported from industrial wastewater, coastal aquifers, and ultimately seawater^6^. While adsorbents applied in wastewater treatment have been considered a suitable technological means for heavy metal removal, challenges nevertheless continue to remain^7^. Carbon-based materials (e.g. activated carbon and carbon nanotubes) synthesized from sustainable lignocellulosic residues have shown to be a particular area of promise within such efforts^8,9^. Likewise, anaerobic digestate from food waste in combination with sodium silicate binder has been used to produce biochar, with Pb removal capacity six times greater than commercial activated carbon being found^10^. Overall, bioadsorbents are found to be effective and safe, representing low-cost alternatives for water treatment.

Consideration of solutions requires an ability to monitor their effectiveness. Accordingly, with the considerable ability of macroalgae to efficiently bioaccumulate heavy metals, macroalgae are considered valuable bioindicators of heavy metals contamination^2,11^.

Macroalgae are aquatic organisms that are present in almost all marine ecosystems^12^. In particular, macroalgae have high reproduction rates, leading to their high abundance and distribution in coastal environments^13^. They can be classified into three large groups: brown algae (Phaeophyceae), red algae (Rhodophyta), and green algae (Chlorophyceae). As examples of potentialities herein, Phaeophyceae, especially the order Fucales, can thrive in waters with high levels of heavy metals^14^. Likewise, green algae, especially the Ulvales order, are great bioindicators due to their high affinity with manganese (Mn), iron (Fe), copper (Cu), zinc (Zn), and lead (Pb)^15^.

In other respects, including high lipid productivity, carbon dioxide capture, and low land requirement, these favor discussion of the suitability of algal biomass in biofuel production^16,17^. Of the several challenges that remain to be addressed, these include algae post-processing and cultivation processes^18^. Several algae species are able to produce bio-oil, as for example via catalytic pyrolysis involving gasification, also liquefaction processes^19^, the former being preferred due to their simplicity, high yields, and straightforward operations^20^.

It is also to be appreciated that many macroalgae are destined for human consumption as a result of their high nutritional value. For instance, *Porphyra* sp. (commercially known as Nori) is frequently consumed worldwide including in the Japanese delicacy “sushi”. Also, among the brown algae, *Laminaria* spp. (Kombu), *Undaria pinnatifida* (Wakame) and *Hizikia fusiforme* (Hiziki) find considerable use in modern European and Asian cuisine^21^. Despite the presence of micronutrients in foodstuffs that are essential for human health, the trace elements have the potential to become highly toxic if certain levels are exceeded^22,23^. A review of the literature reveals that a considerable number of studies throughout the world have shown a greater concentration of heavy metals in seaweed. Pan et al. observed the high bioaccumulation capability of Cu, Cr, Ni, Zn, Pb, Cd, and As by seaweed collected from the Dongtou Islands of the East China Sea^24^. Dadolahi-Sohrab et al. reported elevated concentrations of Pb, Cd, Cu, Ni, Zn, and Fe metals in 11 dominant seaweed species from the Strait of Hormuz^25^. A relatively high concentration of Fe and Pb in green and brown seaweeds collected from the Antikyra Gulf (Viotia, Greece) has been reported by Malea et al.^26^. Besada et al. showed that most edible algae contain elevated levels of cadmium with respect to the concentration limits suggested by European legislation^27^. Specifically, *Hizikia fusiforme* showed the highest concentrations of arsenic (As), which could pose a potential threat to human health. Arulkumar et al. determined very high concentrations of Zn and Cu in *Ulva Lactuca* samples from within India^28^. All of the aforementioned studies indicate that macroalgae have a great capability for bioaccumulating, storing, and persisting in retaining heavy metals and other trace elements, accordingly representing a potential threat to both local fauna and human health^29^.

Bangladesh is a low-lying, riverine South Asia country, with a coastline of 580 km, located on the northern littoral of the Bay of Bengal. The coastal area, with both sandy and muddy beaches, estuaries, and mangrove swamps, provides a favourable habitat for various kinds of seaweed. As a result, in the coastal waters of Cox’s Bazar and the Sundarbans regions more than 133 species of seaweed grow naturally with 14 of these having commercial value^30^. While it has been reported that some 5,000 metric tons of seaweed biomass are annually available in the coastal waters of Bangladesh^31^, nevertheless there is a lack of detailed information on the seaweeds production, distribution, availability of the commercially important species, and approaches for utilization in Bangladesh^32^. Of note is that the commercial value of seaweed is relatively unknown to the majority of Bangladeshi nationals, the numbers of individuals involved in seaweed cultivation in the south-eastern and south-western coasts of Bangladesh being limited. In particular, a small group in the Cox’s Bazar region are noted to be occupied in the collection of seaweed in support of their livelihood, with additional involvement in the export of the medium to Myanmar, China, and Singapore^33^. Tribal populations within the country are also known to be using seaweed as a regular item within their dietary habit. Moreover, seaweed harvested in the country is also being used in the production of a wide range of items, including food, medicine, cosmetics, fertilizers, biofuels, and products to prevent environmental pollution^33^. However, to be the best knowledge of the authors, no earlier published studies are available concerning the quality or metal levels in the macroalgae found in the Cox’s Bazar region.

Acknowledging the problems of seaweed contaminated by heavy metals and trace elements, effective monitoring of these contaminants is necessary, especially in respect of those regions within which high consumption of seaweed is known to be taking place.

While previous studies have shown bioaccumulation rates to differ among macroalgae species, characterization of this within the country is still lacking. Accordingly, the objectives of the present study have been to quantify the concentration of trace elements and heavy metals in different macroalgae species collected from the coastal waters of Cox’s Bazar region and Saint Martin’s Island, also to determine if the macroalgae are more likely to bioaccumulate trace elements based on their species or family (Rhodophyta, Chlorophyta, and Phaeophyceae). The results presented in this study offer such insights, providing groundwork in the biomonitoring of specific algae for heavy metals contamination. Moreover, human health hazard risks (carcinogenic and noncarcinogenic) have also been calculated for the heavy metals that have been found herein to be manifesting at potentially toxic levels, account being taking of consumption behavior.

## Materials and methods

### Study site and sampling

**Figure 1:**
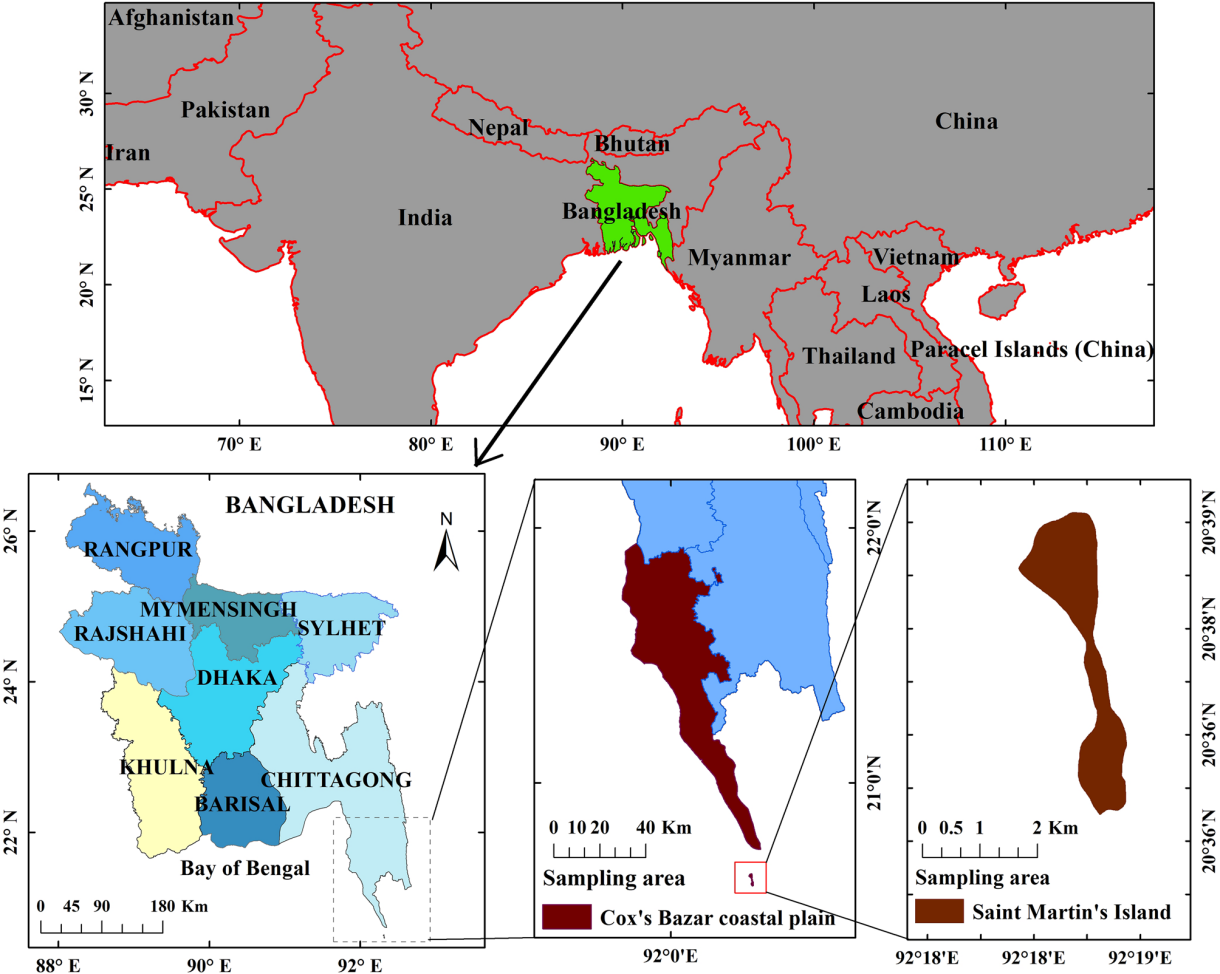
Map of the sampling locations in Cox’s Bazar and Saint Martin’s Island. The map was constructed using ArcGIS 10.7.

The coastal region of Cox’s Bazar and Saint Martin’s Island, along the northern littoral of the Bay of Bengal (Fig. 1) have been selected for sample collection. The coastline of Cox’s Bazar is the longest sandy beach in the world (125 km), including natural landscapes, tertiary hills, sand dunes, etc., all making the location highly attractive, typically with millions of tourists visiting the area annually, with multiple businesses associated with the tourism industry present along the coastline area^34^.

Over the period January 2018 to April 2019, ten naturally growing seaweed species samples were collected (Table 1), each in triplicate, obtained from different sites in the Cox’s Bazar and Saint Martin region of Chittagong (Fig. 1). Species and taxonomy identification followed standard morphological features, size, shape, color, etc. The samples were thoroughly rinsed to remove adhered sediments and other substances. To mitigate against the effects of natural decay and consequent influence on metals analysis, the samples in labeled plastic bags were quickly transported to the lab for subsequent processing.

**Table 1:** Identity and labeling of the 10 seaweed species collected. “R”, “B”, and “G” indicate red, brown, and green seaweed respectively.

Sample **c**ode	Seaweed **s**pecies	Family
R1	*Hypnea musciformis*	Rhodophyta
R2	*Hypnea pannosa*	Rhodophyta
R3	*Jania Rubens*	Rhodophyta
R4	*Gelidium pusillum*	Rhodophyta
B5	*Padina tetrastromatica*	Phaeophyta
B6	*Sargassum oligocystum*	Phaeophyta
B7	*Padina boryana*	Phaeophyta
G8	*Caulerpa racemosa*	Chlorophyta
G9	*Enteromorpha intestinalis*	Chlorophyta
G10	*Ulva compressa*	Chlorophyta

### Sample preparation

The procedure described by Jolly et al. was adopted in preparing the samples for analysis, the latter via the technique of Energy Dispersive X-ray Fluorescence (EDXRF), in the Atmospheric and Environmental Chemistry Laboratory of the Atomic Energy Centre, Dhaka^35^. In specific terms, the preparation procedures consisted of cutting seaweed samples into small pieces, washed with tap water, and subsequently rinsed with deionized water, to then be left to dry at room temperature, followed by placement in an oven at 60 °C until a constant dry weight was obtained. The dried seaweeds were then ground to obtain a fine homogenous powder using an agate mortar and pestle, with 0.1 g masses being pelletized using a hydraulic press pellet maker model (Specac Ltd., UK) by applying a pressure of approximately 3 tons. The dimension of the prepared pellets was 7 mm in diameter and 1 mm thick. This procedure was carried out for each triplicate of the 10 seaweed species. The pellets were then stored in clean glass Petri dishes, then held in a vacuum desiccator for subsequent measurement of the concentrations of the various metals of concern.

### Sample analysis and method validation of the level of concern metals

The elemental concentration was analyzed by energy-dispersive X-ray fluorescence (EDXRF) spectroscopy, as described in our previous study^36^. In brief, a ^109^Cd point source with an X-ray beam (at 22.4 keV) was applied to excite the prepared samples and produce characteristic X-ray detected by the Si(Li) detector (Canberra™) which has a resolution of 175 eV at 5.9 keV, amplified by the spectroscopy amplifier and processed by the multichannel analyzer MCA (6 K + channel). All the peak areas were integrated by AXIL and PRO/QXAS software provided by the International Atomic Energy Agency (IAEA), Vienna, Austria.

With EDXRF a direct comparison method for elemental concentration measurement, calibration curves must be constructed based on similar matrices. Improving the sensitivity of readings and nullifying the matrix effects, the calibration curves were constructed via the use of the commercially available standard reference material (SRM) Orchard leaf/NIST 1571. The average peak areas of the EDXRF irradiated SRM pellets (at least three pellets) prepared using a similar configuration to that of the algae samples were then plotted in terms of the presence of the elements as a function of atomic number^36^. Validation of the calibration curve constructed for elements present in the standards was performed via analysis of another standard reference material, Spinach/NIST 1570a, again prepared in the same way as the algae sample^35^. All the obtained values were similar to certified values, the percentage relative error in evaluated elements being < 10%, assuring validation of the method. Comparison between the experimental and certified values is provided in Table 2.

**Table 2:** Comparison of measurement and certified values (mg kg^−1^, dry weight of CRM Spinach/NIST 1570a.

Element	Results obtained (mg kg^−1^)	± SD	Certified values (mg kg^−1^)	Relative error (%)	CV (%)	Recovery (%)
K	27,354.7	840.1	29,030	5.77	3.07	94.2
Mn	78.62	2.27	75.90	− 3.59	2.89	103.6
^a^Fe	325.67	22.30	300	− 8.56	6.76	108.6
Cu	13.24	0.44	12.20	− 8.56	3.39	108.6
Zn	84.11	1.85	82.00	− 2.57	2.20	102.6
^a^As	9.13	0.21	10.00	8.67	2.28	91.3
Sr	55.21	0.41	55.60	0.71	0.74	99.3
^a^Pb	40.38	1.14	45.00	10.26	2.82	89.7

^a^Certified reference material Orchard leaf/NIST 1571.

Samples were positioned in the EDXRF spectrometer according to the defined geometry and then excited using a ^109^Cd point source, providing 22.4 keV X-rays. To obtain good counting statistics, each sample was irradiated for a sufficient duration, ranging from 2000 to 5000 s. The collected spectra were analyzed using the aforementioned software, obtained from the IAEA. In this study, the standard addition method was used to obtain the metal concentration. The method involves the addition of known quantities of various analytes to the specimen. This method requires a linear calibration throughout the range of addition of various analytes. To determine each of the elements in the obtained spectrum, use was made of the calibration curves, also acknowledging an absence of inter-elemental effects; determination of metal concentrations in the analyzed algae sample was made via Eq. (1) as follows:

1 $$I_{i} = C_{i} \cdot S_{i} \cdot A$$

with *I*_*i*_ the characteristic X-ray net intensity (in cps), *C*_*i*_ the metal concentration (in μg.g^−1^), *S*_*i*_ the sensitivity of each analyzed element *i* (cps·g^−1^·cm^2^), and A the absorption factor, equal to 1 for the samples, prepared in a thin-film geometry.

### Determination of the limit of detection

The Minimum Detection Limit (MDL) depends on the counting statistics of the measurement and is a statistical process^37^. The MDL is obtained from the ratio of the amount of an element (in ppm) yielding an X-ray intensity equal to 3σ of the background under the peak in an interval equal to the full width at half maximum (FWHM) of the peak and the concentration of the corresponding elements determined by using the calibration procedure^38^, and calculated using relation (2):

2 $$MDL \left( x \right) = \frac{{3{\upsigma }\;{\text{counts}}\;{\text{of }}\;{\text{element }}\;{\hbox{'x'}}\;{\text{ in}}\;{\text{ the}}\;{\text{ sample}}}}{{\frac{{{\text{Counts}}}}{{{\text{ppm}}}}{\text{ of }}\;{\text{element}}\;{\hbox{'x'}}\;{\text{ in}}\;{\text{ the}}\;{\text{ standard}}}}$$

where $\sigma = \sqrt {\frac{Background}{{Channel}} \times FWHM}$ of the relevant element.

The calculated MDL for the analyzed elements is displayed in Table 3.

**Table 3:** MDL values (mg kg^−1^) of most analyzed elements.

Element	MDL (mg kg^**−**1^)
K	8.09
Cr	0.29
Mn	0.27
Fe	0.54
Co	0.17
Cu	0.13
Zn	0.35
As	0.02
Sr	0.19
Pb	0.12

### Metal pollution index (MPI)

The heavy metal burden of the 10 seaweed species was estimated on the basis of a metal pollution index (MPI), calculated using the following formula^39^:

3 $$MPI = \left( {M_{1} \times M_{2} \times \ldots \times M_{n} } \right)^{1/n}$$

with $M_{n}$ the mean concentration of heavy metal n (mg kg^−1^ dry weight). The metals included in the analyses were As, Pb, and Cr.

### Health risk assessment

#### Non-carcinogenic risk

A health risk assessment for an average adult was conducted following Ref.^28^, based on the method of the US Enviromental Protection Agency (EPA)^40^. The targeted hazard quotient (THQ) and hazard index (HI) was calculated based on the exposed dose (ED), the latter calculated using the following Eq. (4):

4 $$ED = \frac{{C_{i} \times D_{i} \times E_{d} }}{{B_{w} \times A_{t} }}$$

with C_i_ the mean concentration of heavy metals in seaweed (mg kg^−1^), D_i_ the daily seaweed intake (5.2 g person^-1^ day^−1^), E_d_ the average duration of exposure (70 years), B_w_ the average body weight of the consumer (70 kg), and A_t_ the lifetime of the consumer (70 years), the values being from recognized reference data.

The THQ, characterizing the non-carcinogenic risk to an exposed individual, is defined as the ratio of the exposed dose of a particular metal to the corresponding reference dose (R_f_D) and can be determined by the following Eq. (5):

5 $$THQ = \frac{ED}{{R_{f}D}}$$

where R_f_D is the recommended oral reference dose for certain metals. Lastly, hazard index (HI) is calculated following Eq. (6):

6 $$HI = \sum THQ$$

Following the US EPA^40^ guidelines, a HI < 1 is regarded to offer no potential health risk.

#### Carcinogenic risk

The carcinogenic risk (CR) was assessed following Kortei et al. based on the methodology by the US EPA^41^, using Eq. (7)^42^:

7 $$CR = ED \times CSF$$

where CSF is the cancer slope factor. The CSF values of carcinogenic metals are displayed in Table 4. The total cancer risk (CR_t_) was determined as the sum of the CR from the studied heavy metals^43^, as displayed in Eq. (8):

8 $$CR_{t} = \sum CR$$

**Table 4:** Cancer slope factor values for As, Pb, and Cd.

Metal	CSF (mg kg^−1^ day^−1^)	References
As	1.5	^43^
Pb	8.5 × 10^−3^	^42^
Cr	0.5	^43^

### Statistical analysis

Metal concentrations in seaweed were expressed in mg/kg ± standard error of the mean (mg kg^−1^ ± SEM). To allow meaningful comparison, dry weight (d.w.) concentrations were converted to wet weight (w.w.) values using the simple formula^37^:

9 $$Concentration \left( {w.w.} \right) = \frac{{\left( {100 -\%\; of\;water\; in\; seaweed} \right)}}{100} \times Concentration \left( {d.w.} \right)$$

Given that seaweed generally contains 80–90% water^44^, a median value of 85% was used for the calculations.

An ordinary one-way analysis of the variance (ANOVA) was conducted to compare the concentrations of the 13 observed elements among the 10 seaweed species, followed by Tukey’s multiple comparisons test. The same analyses were used to compare the concentration of the elements among types (Chlorophyta, Rhodophyta, and Phaeophyceae) by grouping seaweed species of the same color. Statistical significance was set to 0.05. All the analyses and graphs were performed in GraphPad Prism (version 8.4.3 for Windows).

## Results and discussion

A total of 13 elements were determined and validated by the EDXRF technique for the 10 macroalgae species. Overall, the mean concentration of trace elements and heavy metals in the 10 species decreased in the descending order K > Fe > Zr > Br > Sr > Zn > Mn > Rb > Cu > As > Pb > Cr > Co (Table 5). The mean elemental concentration and descriptive statistics per macroalgae species are displayed in Table S1. As expected, K provides the greatest concentration among the 10 species (mean of 4.2 × 10^4^ mg kg^−1^), being significantly greater than the second most prevalent element (Fe, with a mean of 1.9 × 10^3^ mg kg^−1^). Previous studies have shown the prevalence of K to be in the same order. For instance, in Ulva spp. (Ireland), *Chondrus crispus* (Denmark), and *Fucus spiralis* (Spain), the mean K respective concentrations were 1.2 × 10^4^, 3.3 × 10^4^, and 4.0 × 10^4^ mg kg^−1^^45–47^. Seaweeds naturally contain high K content, at of the order of 2% of their dry weight^48,49^, depending on the species and environmental conditions. Co concentrations have been found to be the lowest in value, at a mean of 0.26 mg kg^−1^ (ranging from 0.18 to 0.33 mg kg^−1^ across the species); lower Co content has been reported in commercial seaweed of Asian origin (mean of 0.10 mg kg^−1^) and European origin (mean of 0.03 mg kg^−1^)^50^, constituting a naturally occurring micronutrient in seaweed^51^. Previous studies in aqueous solutions have demonstrated the leaching of elements such as Ca and Mg from grasses/algae, directly measured via inductively coupled plasma mass spectrometry (ICP-MS)^52^. In the present study, macroalgae samples were not analyzed in aqueous solutions, thus leaching and loss of metallic elements from the samples being unlikely to have occurred.

**Table 5:** Mean concentrations of trace elements and heavy metals in the 10 species of algae collected from the coastal waters of the Cox’s Bazar region.

Element	Concentration	
	Wet weight	Dry weight
K	41,234.0	6185.1
Cr	1.15	0.17
Mn	32.9	4.9
Fe	1971.5	295.7
Co	0.26	0.04
Cu	16.6	2.5
Zn	34.5	5.2
As	3.4	0.50
Br	63.7	9.6
Rb	28.0	4.2
Sr	39.8	6.0
Zr	78.5	11.8
Pb	2.6	0.39

**Figure 2:**
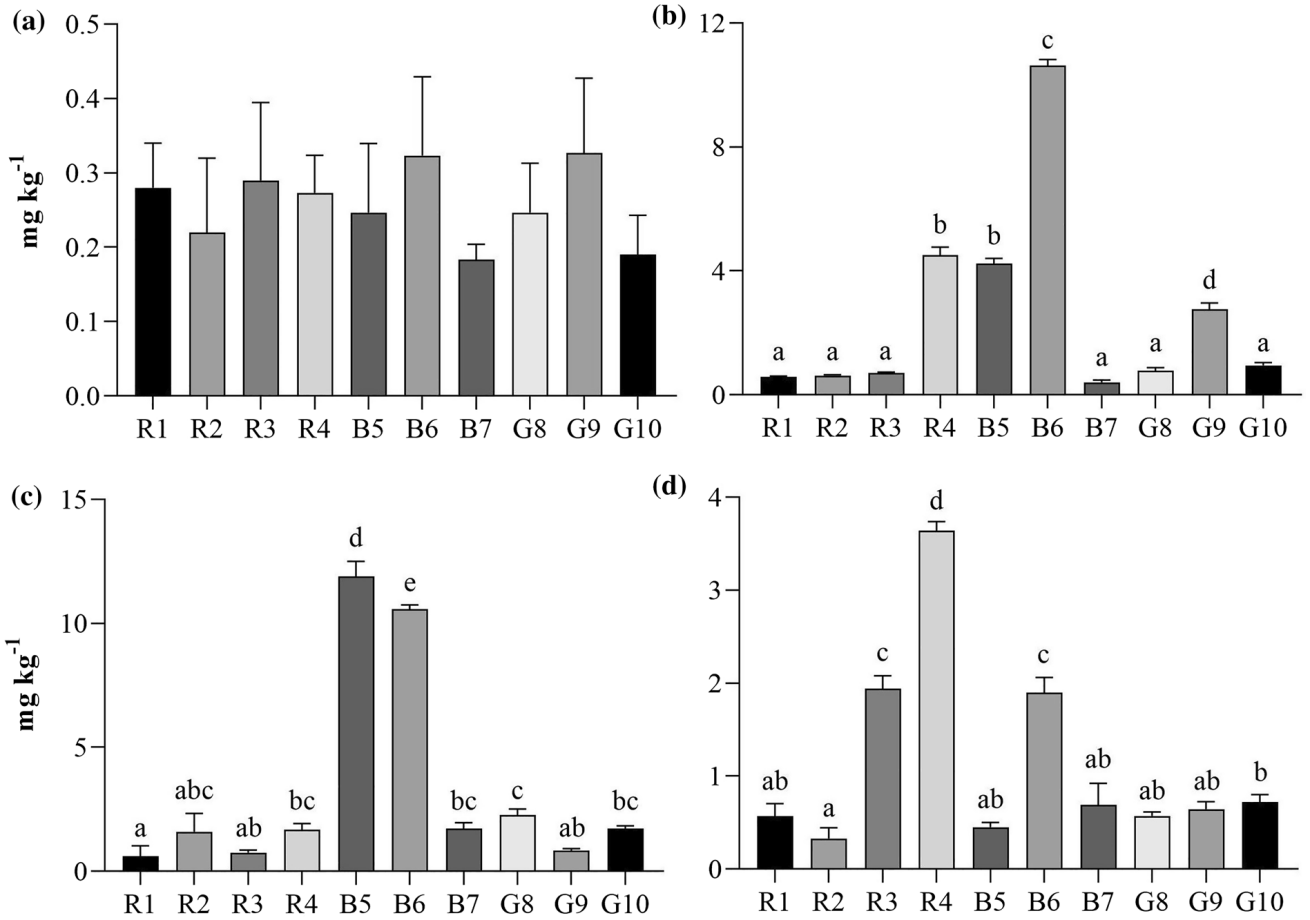
Mean concentration of (**a**) Co, (**b**) Pb, (**c**) As, and (**d**) Cr in the 10 algae species evaluated. Error bars indicate standard deviation. Letters indicate significant differences.

One-way ANOVA analysis shows a significant difference in the concentration of elements among macroalgae species (*p* =  < 0.05), except for Co (*p* = 0.3669) (Fig. 2a). Regarding the determined hazardous heavy metals, Pb, As, and Cr; the highest mean concentrations of Pb were found in B6 (10.63 mg kg^−1^), followed by R4 (4.50 mg kg^−1^), and B5 (4.24 mg kg^−1^) (Fig. 2b). For arsenic, two species, B5 (11.89 mg kg^−1^) and B6 (10.75 mg kg^−1^) had significantly higher concentrations than the rest (Fig. 2c). Lastly, R4 (3.64 mg kg^−1^), R3 (1.94 mg kg^−1^), and B6 (1.90 mg kg^−1^) presented the highest levels of Cr (Fig. 2d). Interestingly, macroalgae species from the same genus (*Hypnea*; R1 and R2) showed similar bioaccumulation of heavy metals. B5 and B7 (both genius *Padina*), showed considerable differences. This suggests that despite similar mechanisms of uptake, total nutrient/metal uptake may be species-specific for some seaweed genera.

Despite being consumed as food or utilized as animal feed, there is no current legislation in Bangladesh that determines the maximum levels of heavy metals in seaweed. However, some international norms are available. Pb concentration in B6 surpassed the maximum levels in seaweeds (5 mg kg^−1^) recommended by the French High Council for Public Health^53^ and The Center for the Study and Development of Algae (CEVA)^28^. Moreover, the maximum levels of Pb in leafy vegetables (which may be consumed at similar levels to that of seaweed), according to the FAO^54^, is much lower (at 0.3 mg kg^−1^). To the best of our knowledge, maximum As and Cr levels in seaweed have not been addressed in international regulations. It should be noted that *Sargassum sp.* along with four additional species in the present study (R2*,* R4*,* G8*,* and G9) have been cultured and consumed in fresh or dried form for decades in Bangladesh^32^, thus presenting a potential route for heavy metal exposure to the population. Nevertheless, seaweed cultivation in Cox’s Bazar remains artisanal and undeveloped^31^.

The concentrations of the three potentially toxic heavy metals that have been considered herein, Cr, As, and Pb, have been found comparable or lower than literature data elsewhere (Table 6). For instance, the mean concentration of Pb in 12 algal species in China was 1.89 mg kg^−1^ (ranging from 0.77 to 4.21 mg kg^−1^)^24^. In Lebanon, concentrations were even lower, with a mean of 1.04 mg kg^−1^^55^. Cr and As were particularly high in Greece and China, with mean concentrations of 9.38 and 18.33 mg kg^−1^ respectively^1,24,56^. Conversely, much lower concentrations were reported from a market survey in Italy^50^. The mean concentrations of Cr, As, and Pb were 0.14, 1.42, and 0.13 mg kg^−1^, respectively. This may be attributed to the high hygiene standards through the food supply chain and also during seaweed culture intended for human consumption. In markets from the Canary Islands (of Spain) reported Pb concentrations in seaweeds of Asian and European origin have ranged from 0.12 to 0.004 mg kg^−1^ and from < LOQ to 0.05 mg kg^−1^, respectively^57^. The importation of potentially contaminated edibles as a source of heavy metal exposure has been discussed by others, including in regard to the consumption of seaweed. Particular examples include toxic industrial discharges in India, with toxic heavy metal pollution in areas of plant cultivation being a particular consequence^58^. A recent study carried out along the Palk Bay coast of southeast India has observed highly elevated Pb concentrations in many seaweed species, surpassing 10 mg kg^−1^ in many cases. The variation in concentrations was found to depend on the sampling season. The main sources of contamination have been linked to ship washing activities, seafood processing, domestic sewage, and effluent discharges. In the case of boat maintenance procedures, the application of antifouling paint has been noted, the particles of these containing metallic-based biocides that can detach from the marine coatings^59^.

**Table 6:** The concentration of potentially toxic heavy metals in various studies.

Country of study	Species	Tissue	Cr	As	Pb	Ref
Bangladesh	*Hypnea musciformis*	Thalli	0.57 ± 0.08	0.60 ± 0.25	0.59 ± 0.01	This study
	*Hypnea pannosa*		0.33 ± 0.07	1.60 ± 0.43	0.62 ± 0.02	
	*Jania rubens*		1.94 ± 0.08	0.76 ± 0.06	0.71 ± 0.01	
	*Gelidium pusillum*		3.64 ± 0.06	1.68 ± 0.14	4.50 ± 0.15	
	*Padina tetrastromatica*		0.45 ± 0.03	11.89 ± 0.35	4.24 ± 0.10	
	*Sargassum oligocystum*		1.90 ± 0.09	10.57 ± 0.10	10.63 ± 0.11	
	*Padina boryana*		0.69 ± 0.13	1.70 ± 0.14	0.40 ± 0.04	
	*Caulerpa racemose*		0.57 ± 0.03	2.27 ± 0.14	0.77 ± 0.06	
	*Enteromorpha intestinalis*		0.64 ± 0.05	0.84 ± 0.05	2.76 ± 0.12	
	*Ulva compressa*		0.72 ± 0.04	1.72 ± 0.07	0.95 ± 0.05	
Greece	*Gracilaria gracilis*	Thalli	3.89 ± 1.18	4.46 ± 1.07	4.24 ± 1.54	^56^
	*Codium fragile*		10.46 ± 4.84	4.25 ± 0.49	3.89 ± 2.02	
	*Ulva intestinalis*		13.80 ± 2.96	1.50 ± 0.47	4.62 ± 1.19	
	*Ulva ridiga*		9.38 ± 1.50	1.45 ± 0.25	3.06 ± 0.67	
Lebanon	*Ulva lactuca*	N.S	1.08 ± 0.90	4.78 ± 3.60	1.04 ± 1.03	^55^
China	*Sargassum fusiforme*	N.S	0.85 ± 0.11	57.71 ± 13.44	1.50 ± 0.62	^24^
	*Sargassum thunbergii*		3.84 ± 0.47	49.08 ± 2.46	2.00 ± 0.24	
	*Sargassum vachellianum*		1.21 ± 0.12	23.77 ± 3.88	1.90 ± 0.39	
	*Pachydictyon coriaceum*		5.90	16.12	3.34	
	*Polyopes polyideoides*		0.92 ± 0.25	15.41 ± 0.05	1.32 ± 0.11	
	*Gelidium divaricatum*		6.83 ± 1.11	8.89 ± 1.07	4.21 ± 0.64	
	*Gracilaria lemaneiformis*		0.86	8.31	0.90	
	*Ahnfeltiopsis flabelliformis*		1.16 ± 0.03	4.81 ± 0.74	1.09 ± 0.09	
	*Laurencia tropica*		3.01 ± 0.18	9.72 ± 2.70	1.92 ± 0.19	
	*Pterocladiella capillacea*		1.89 ± 0.23	3.35 ± 0.23	1.58 ± 0.04	
	*Chondracanthus intermedius*		1.04 ± 0.07	15.41 ± 0.25	0.77 ± 0.12	
	*Ulva pertusa*		2.05	7.35	2.18	
Italy	*Himanthalia sp.*	N.S	0.1 ± 0.04	0.39 ± 0.51	0.06 ± 0.03	^50^
	*Saccharina sp.*		0.08 ± 0.03	0.87 ± 0.88	0.17 ± 0.39	
	*Undaria sp.*		0.12 ± 0.06	1.37 ± 1.64	0.10 ± 0.07	
	*Ascophyllum sp.*		0.23	0.09	0.11	
	*Laminaria sp.*		0.13	7.14	0.11	
	*Porphyra sp.*		0.08 ± 0.10	1.27 ± 1.37	0.17 ± 0.30	
	*Palmaria sp.*		0.11 ± 0.06	0.09 ± 0.05	0.14 ± 0.21	
	*Ulva sp.*		0.30 ± 0.18	0.12 ± 0.09	0.16 ± 0.12	
China	*Porphyra sp.*	N.S	1.64 ± 0.08	36.67 ± 0.53	0.96 ± 0.03	^79^
	*Laminaria sp.*		3.78 ± 0.56	43.85 ± 1.42	0.61 ± 0.03	

*N.S.* Not Specified.

**Figure 3:**
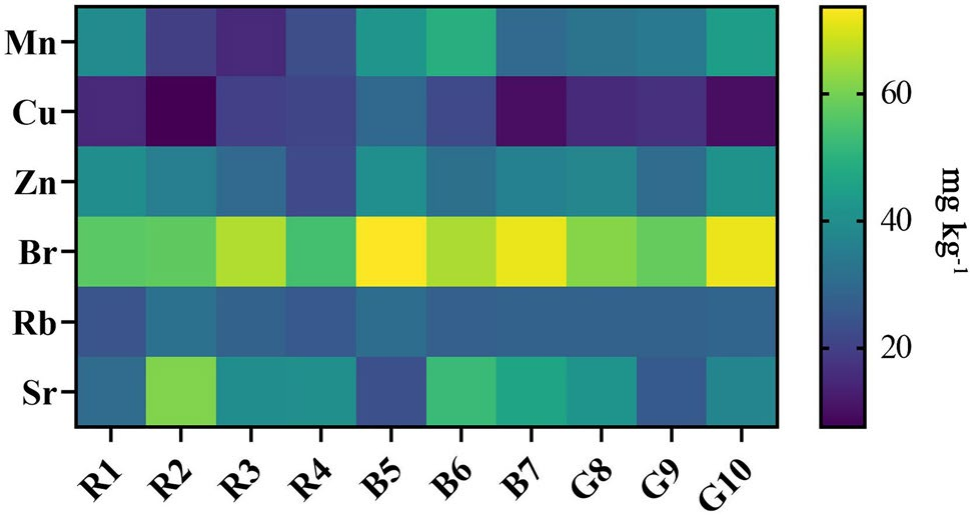
Heat map showing the concentrations (ranging from ~ 7.8 to 73.6 mg kg^−1^) of six elements found within the 10 algae species.

The concentrations of most of the remaining trace elements (Mn, Cu, Zn, Br, Rb, and Sr) have been found to be in the range ~ 5 to 75 mg kg^−1^ (Fig. 3). Additionally, the mean Fe ranged from 1487 mg kg^−1^ in *P. tetrastromatica* to 3352 mg kg^−1^ in *H. pannosa*. Most of these elements are micronutrients involved in natural algae metabolism^60^. The concentration of micronutrients found in the present study is mostly of the same order of magnitude as that reported by Roleda et al.^61^. Seaweeds in particular are considered a great source of Fe^62^, which could increase the proportion of absorbed iron in functional meals^63^. Sr is known to be related to cell wall polysaccharides found in some macroalgae, such as alginates in most Phaeophyceae, and generally exhibit low concentrations^64^. Although Rb is not widely studied, it is suggested to be related to the geochemistry of the coastal environments^65^. Algae are noted to use the bromine and chlorine present in the environment to biosynthesize halogenated secondary metabolites^66^, many of the halogenated compounds being found to be brominated.

Concerning seaweed type, most of the studied metals show insignificant differences except for Mn, Fe, As, Br, Zr, and Pb (Table 7). The results of the Kruskal–Wallis test and literature suggest that the seaweed type may not be as significant as the species in determining the bioaccumulation of metals in the seaweed. For instance, Rubio et al. analyzed 20 metals, finding no significant differences between Phaeophyta and Rhodophyta for the majority of the studied metals, the exceptions being Cr, Cu, Fe, Li, Mn, Mo, Sr, V, and Zn^67^. In contrast, Filippini et al. performed a similar analysis with 21 metals comparing Phaeophyta, Rhodophyta, and Chlorophyta, finding significant differences in the majority of studied metals, the exceptions being for Pb, Hg, Mn, Co, Ti, and Sb^50^. Some comparative studies have pointed to Phaeophyta being the most efficient algae in accumulating metals^68^, while others have pointed to Rhodophyta^24,50,69^. These results suggest that the influence of algae type in metal bioaccumulation may be limited, while some specific characteristics such as surface area and growth rates may be more important^70^. As apparent in Table 6, the specific macroalgae tissue evaluated in recent studies has not generally been specified^55,56^. Of note is that heavy metal and trace element concentrations have been observed to vary significantly in macrophytes from salt marshes, specifically between roots, shoots, and leaves^36^. Sáez et al. studied the bioaccumulation of metals in the thallus (blade, stipe, and holdfast) of the kelp *Lessonia trabeculata* ^71^, finding metal-specific affinity in certain parts. A further variable not taken into account has been the life stage of the selected organisms. Future investigations should focus on determining the influence of the particular tissue and of seaweed age on the concentration of heavy metals.

**Table 7:** Overall mean elemental concentration per group of algae and ANOVA results.

Element	Mean (mg kg^−1^) ± SEM per algae group			ANOVA results	
	Rhodophyta	Phaeophyta	Chlorophyta	F (2, 27)	*P* value
K	40,836 ± 2172	42,971 ± 1412	40,027 ± 1061	0.6717	0.5192
Cr	1.62 ± 0.40	1.01 ± 0.23	0.646 ± 0.030	2.769	0.0805
Mn	24.2 ± 3.3	40.4 ± 2.9	37.0 ± 1.9	9.179	0.0009
Fe	2475 ± 160	1634 ± 63	1638 ± 55	17.63	< 0.0001
Co	0.266 ± 0.022	0.251 ± 0.031	0.254 ± 0.030	0.08797	0.9160
Cu	15.8 ± 1.6	20.6 ± 2.9	13.8 ± 1.1	2.919	0.0711
Zn	32.2 ± 2.0	36.5 ± 1.3	37.3 ± 1.5	2.672	0.0873
As	1.16 ± 0.18	8.06 ± 1.60	1.61 ± 0.21	19.97	< 0.0001
Br	58.6 ± 1.4	70.3 ± 1.2	63.9 ± 2.1	14.50	< 0.0001
Rb	27.7 ± 0.9	28.8 ± 0.6	28.2 ± 0.1	0.5986	0.05567
Sr	43.0 ± 3.4	40.5 ± 4.4	34.9 ± 2.4	1.382	0.2684
Zr	116.2 ± 13.9	45.6 ± 1.0	61.3 ± 9.6	12.43	0.0001
Pb	1.61 ± 0.51	5.09 ± 1.49	1.50 ± 0.32	5.276	0.0116

Algae-based heavy metal monitoring may require the use of different species that have better bioaccumulation affinity for different metals. For instance, the highest As concentrations in 12 macroalgae species from China were found in three species from the genus Sargassum^24^. Accordingly, in the present study, *S. oligocystum* was the species that exhibited the second highest As concentration (10.6 mg kg^−1^), slightly below *P. tetrastromatica* (11.9 mg kg^−1^), and significantly greater than the third-highest (*C. racemose*; 2.27 mg kg^−1^). Similarly, in the present study the highest Cr concentrations were found in species from the genus Gelidium, also as reported by Pan et al.^72^. The consistency of our results with previous studies supports the necessity of determining genius-specific macroalgae for heavy metal monitoring. Based on our results, we suggest the genus Gelidium for Cr, Padina for Cu and As, and Sargassum for Mn, As, and Pb in monitoring. Future studies may determine the uptake routes for different heavy metals in macroalgae based on their botanical characteristics.

**Figure 4:**
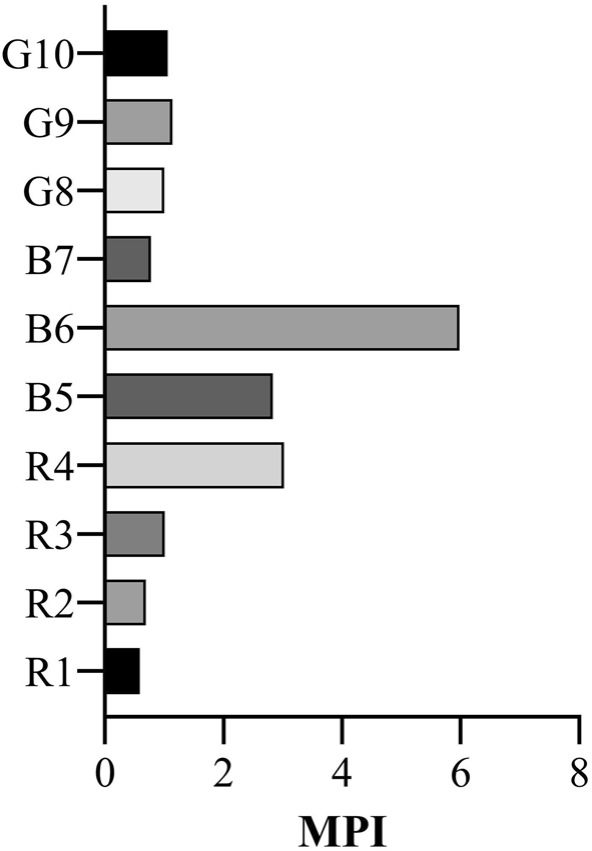
Metal pollution index (MPI) for the ten seaweed species investigated.

The results of the carcinogenic and non-carcinogenic health risk assessment are displayed in Table 8, while MPI values of the 10 seaweed species are summarized in Fig. 4. The six metals, Cr, Pb, Cu, Zn, Co, and As, were selected for their potentially hazardous nature at relatively high concentrations and oral R_f_D data availability. The overall Hazard index was 0.993, which means the evaluated metals together may not pose a serious health risk to human health. However, Cd and Hg were not evaluated in the present study. These heavy metals could potentially contribute to a targeted hazard quotient sufficient to reach a HI > 1, thus representing a moderate to high risk for adverse human health effects. In the case of the carcinogenic risk, the CR_t_ was calculated as 0.436. Chen et al.^72^ estimated the HI from dry seaweed consumption in southeastern China by considering the elements Al, Cd, Cr, Cu, Hg, Ni, and Pb. The overall mean HI was calculated as 0.22, well below our estimations. However, the HI calculated for the Malaysian population considerably exceeds the health hazard limit (HI = 4.38), although that study presented a lower CR_t_ than the present study (0.29)^5^. Although many studies concerning specific populations suggest heavy metal consumption from seaweed may not pose a sizeable health hazard^67,73^, in the present regard of Cox’s Bazar the health authorities should look to monitoring heavy metal in consumable seaweeds, taking HI and CR_t_ as indicators of their potential toxicity in areas of high seaweed consumption. In considering the calculated MPI of high toxicity heavy metals (Cr, Pb, and As) among the various seaweed species, it is suggested that B6 (*S. oligocystum*) and R4 (*G. pusillum*) may be the species that present the highest risk of heavy metal ingestion in Cox’s Bazar.

**Table 8:** Carcinogenic and non-carcinogenic health risk assessment from seaweed consumption in adults in Bangladesh.

Metal	Oral R_f_D	Ci	ED	CSF	THQ	CR
Cr	3	1.12	0.083	0.5	0.028	0.042
Pb	3.6	2.59	0.192	8.5 × 10^−3^	0.053	0.002
Cu	37	16.3	1.212	–	0.033	–
Zn	300	34.5	2.560	–	0.009	–
Co	30	0.21	0.016	–	0.001	–
As	0.3	3.51	0.261	1.5	0.870	0.392
					HI = 0.993	CR_t_ = 0.436

Oral R_f_D and CSF values were obtained from Kamuda et al. ^80^, and Kortei et al. ^42^, and Shams et al. ^43^ respectively.

The toxicological implications of exposure to heavy metals are widely understood^74^. As an instance, lead poisoning generally links with anemia, affecting three enzymes associated with heme synthesis. In extremely high exposure scenarios, the neurological system can be critically affected^75^. Hexavalent chromium [Cr(VI)] and As are two well-established heavy metals giving rise to carcinogenic effects^76,77^. Despite the critical health effects associated with the exposure to some of the evaluated heavy metals, seaweed consumption by members of the public remains relatively low, likely insufficient to manifest in severe health effects. Of clinical cases concerning heavy metal tainted foodstuffs, these have mostly been attributed to the consumption of contaminated drinking water^78^.

## Conclusions

Marine macroalgae are regarded as potential biomonitors of heavy metal and trace element pollution. In the present study, the concentrations of 13 elements were determined in 10 species of macroalgae from the coastal area of Cox’s Bazar and Saint Martin’s Island. The elemental bioaccumulation affinity per species and family type was investigated. Results indicate that some species may serve as better biomonitors than others for certain elements. Based on the agreement with the available literature, we suggest the genus Sargassum for Mn, As, and Pb monitoring, Gelidium for Cr, and Padina for Cu, and As. Additionally, since many of the species investigated in the present study are cultivated for human consumption, the relevant hazard indices were determined. The index value remains marginally below the limits recommended. However, since other important toxic heavy metals, such as Cd, were not evaluated in the present study, the index may be underestimated. Future research must focus on determining the botanic implications and biochemical routes that determine the heavy metal bioaccumulation affinity of key species.

## Supplementary Information

Supplementary Information.
