# Supplementary material for: Macroalgae in biomonitoring of metal pollution in the Bay of Bengal coastal waters of Cox’s Bazar and surrounding areas
Source: Sci Rep. 2021 Oct 25;11:20999. doi: 10.1038/s41598-021-99750-7 (PMC8546050; doi:10.1038/s41598-021-99750-7)
Supplement: Supplementary file 1 — Supplementary Information. [file 41598_2021_99750_MOESM1_ESM.docx]

**Macroalgae in biomonitoring of metal pollution in the Bay of Bengal coastal waters of Cox’s Bazar and surrounding areas**

Md. Refat Jahan Rakib^a,*^, Y.N. Jolly^b^, Diana Carolina Dioses-Salinas^c^, Carlos Ivan Pizarro-Ortega^c^, Gabriel Enrique De-la-Torre^c^, Mayeen Uddin Khandaker^d^ , Abdullah Alsubaie^e^, Abdulraheem SA Almalki^f^, D.A. Bradley^d,g^

^a^Department of Fisheries and Marine Science, Faculty of Science, Noakhali Science and Technology University, Noakhali, Bangladesh

^b^Atmospheric and Environmental Chemistry Laboratory, Atomic Energy Centre Dhaka 1000, Bangladesh

^c^Universidad San Ignacio de Loyola, Av. La Fontana 501, Lima 12, Lima, Peru

*^d^Centre for Applied Physics and Radiation Technologies, School of Engineering and Technology, Sunway University, 47500, Bandar Sunway, Selangor, Malaysia*

*^e^Department of Physics, College of Khurma, Taif University, P.O. Box 11099, Taif 21944, Saudi Arabia.*

*^f^Department of Chemistry, Faculty of Science, Taif University, Taif 21974, Saudi Arabia.*

*^g^Department of Physics, University of Surrey, Guildford GU2 7XH, UK*

Supplementary material

**Table S1**. Mean, SD and SEM of the 13 elements analyzed among 10 macroalgae species (dry weight).

|  | Code | R1 | R2 | R3 | R4 | B5 | B6 | B7 | G8 | G9 | G10 |
| --- | --- | --- | --- | --- | --- | --- | --- | --- | --- | --- | --- |
| K | Mean | 35766 | 41727 | 52067 | 33786 | 47513 | 43606 | 37794 | 39221 | 43984 | 36876 |
|  | SD | 2612 | 27.54 | 795.70 | 656.40 | 270.70 | 37.29 | 41.19 | 34.39 | 1081 | 96.54 |
|  | SEM | 1508 | 15.90 | 459.40 | 379 | 156.30 | 21.53 | 23.78 | 19.86 | 624 | 55.74 |
| Cr | Mean | 0.57 | 0.33 | 1.94 | 3.64 | 0.45 | 1.90 | 0.69 | 0.57 | 0.64 | 0.72 |
|  | SD | 0.14 | 0.12 | 0.14 | 0.10 | 0.05 | 0.16 | 0.23 | 0.04 | 0.08 | 0.08 |
|  | SEM | 0.08 | 0.07 | 0.08 | 0.06 | 0.03 | 0.09 | 0.13 | 0.03 | 0.05 | 0.04 |
| Mn | Mean | 39.05 | 19.64 | 15 | 22.91 | 41.97 | 49.15 | 30 | 32.52 | 34.43 | 44.09 |
|  | SD | 14.66 | 0.42 | 5.30 | 1.19 | 1.24 | 2.73 | 1.90 | 2.76 | 1.25 | 1.80 |
|  | SEM | 8.46 | 0.24 | 3.06 | 0.69 | 0.71 | 1.58 | 1.10 | 1.59 | 0.72 | 1.04 |
| Fe | Mean | 2266 | 3352 | 2357 | 1927 | 1487 | 1881 | 1533 | 1572 | 1850 | 1490 |
|  | SD | 27.34 | 37.85 | 29.05 | 17.68 | 29.63 | 23.56 | 46.82 | 32.72 | 36.05 | 4.11 |
|  | SEM | 15.78 | 21.85 | 16.77 | 10.21 | 17.11 | 13.60 | 27.03 | 18.89 | 20.82 | 2.37 |
| Co | Mean | 0.28 | 0.22 | 0.29 | 0.27 | 0.25 | 0.32 | 0.18 | 0.25 | 0.33 | 0.19 |
|  | SD | 0.06 | 0.10 | 0.10 | 0.05 | 0.09 | 0.11 | 0.02 | 0.07 | 0.10 | 0.05 |
|  | SEM | 0.03 | 0.06 | 0.06 | 0.03 | 0.05 | 0.06 | 0.01 | 0.04 | 0.06 | 0.03 |
| Cu | Mean | 14.92 | 7.56 | 19.82 | 20.91 | 29.54 | 22.27 | 9.87 | 15.13 | 16.64 | 9.66 |
|  | SD | 0.13 | 0.45 | 0.15 | 0.91 | 0.23 | 0.65 | 0.16 | 0.45 | 0.31 | 0.10 |
|  | SEM | 0.08 | 0.26 | 0.08 | 0.53 | 0.13 | 0.38 | 0.09 | 0.26 | 0.18 | 0.06 |
| Zn | Mean | 39.59 | 35.12 | 30.12 | 22.23 | 40.23 | 31.42 | 36.24 | 38.14 | 30.45 | 41.12 |
|  | SD | 39.75 | 36.75 | 32.09 | 21.75 | 40.45 | 32.08 | 36.65 | 38.92 | 31.54 | 41.75 |
|  | SEM | 40.12 | 35.55 | 31.05 | 22.65 | 41.75 | 32.75 | 37.02 | 39.05 | 32.45 | 42.08 |
| As | Mean | 0.60 | 1.60 | 0.76 | 1.68 | 11.89 | 10.57 | 1.72 | 2.27 | 0.84 | 1.72 |
|  | SD | 0.43 | 0.74 | 0.11 | 0.25 | 0.61 | 0.17 | 0.24 | 0.25 | 0.08 | 0.12 |
|  | SEM | 0.25 | 0.43 | 0.06 | 0.14 | 0.35 | 0.10 | 0.14 | 0.14 | 0.05 | 0.07 |
| Br | Mean | 56.78 | 57.60 | 65.90 | 53.98 | 73.69 | 65.51 | 71.62 | 61.76 | 58.10 | 71.94 |
|  | SD | 0.31 | 0.47 | 0.24 | 0.90 | 0.38 | 0.42 | 0.41 | 0.29 | 0.14 | 0.10 |
|  | SEM | 0.18 | 0.27 | 0.14 | 0.52 | 0.22 | 0.24 | 0.24 | 0.16 | 0.08 | 0.06 |
| Rb | Mean | 24.65 | 32 | 27.79 | 25.63 | 30.81 | 27.17 | 27.79 | 27.94 | 27.86 | 28.64 |
|  | SD | 24.87 | 32.77 | 27.85 | 26.09 | 31.05 | 27.79 | 27.85 | 28.50 | 27.98 | 28.55 |
|  | SEM | 25.04 | 32.25 | 28.09 | 25.85 | 30.93 | 27.45 | 28.05 | 27.82 | 28.05 | 28.86 |
| Sr | Mean | 30.44 | 61.20 | 39.77 | 40.47 | 23.40 | 52.18 | 45.95 | 41.54 | 25.77 | 37.47 |
|  | SD | 0.67 | 1.38 | 0.57 | 0.86 | 0.71 | 1.52 | 0.76 | 1.06 | 0.31 | 0.64 |
|  | SEM | 0.39 | 0.80 | 0.33 | 0.50 | 0.41 | 0.88 | 0.44 | 0.61 | 0.18 | 0.37 |
| Zr | Mean | 74.75 | 193.80 | 103.60 | 92.67 | 47.35 | 47.65 | 41.86 | 41.81 | 99.48 | 42.45 |
|  | SD | 0.38 | 0.19 | 0.47 | 0.49 | 0.36 | 0.49 | 0.22 | 1.83 | 0.68 | 0.60 |
|  | SEM | 0.22 | 0.11 | 0.27 | 0.28 | 0.21 | 0.28 | 0.13 | 1.06 | 0.39 | 0.34 |
| Pb | Mean | 0.59 | 0.62 | 0.71 | 4.50 | 4.24 | 10.63 | 0.40 | 0.77 | 2.76 | 0.95 |
|  | SD | 0.02 | 0.03 | 0.02 | 0.26 | 0.17 | 0.20 | 0.07 | 0.11 | 0.21 | 0.09 |
|  | SEM | 0.01 | 0.02 | 0.01 | 0.15 | 0.10 | 0.11 | 0.04 | 0.06 | 0.12 | 0.05 |


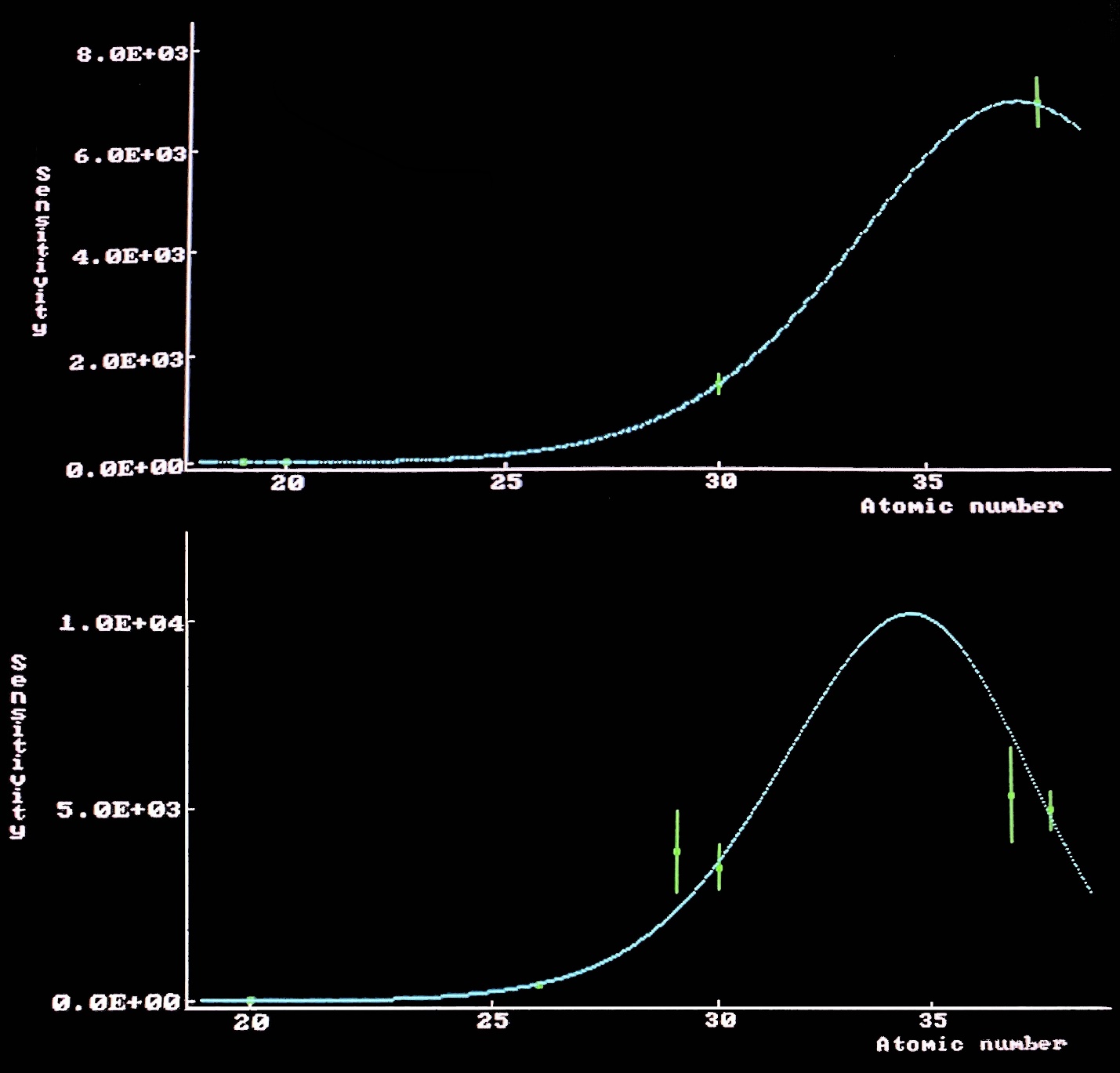


**Fig. S1.** Constructed calibration curves for elemental concentration estimation.
